# Alterations in complex lipids in tumor tissue of patients with colorectal cancer

**DOI:** 10.1186/s12944-021-01512-x

**Published:** 2021-08-04

**Authors:** Alicja Pakiet, Kinga Sikora, Jarek Kobiela, Olga Rostkowska, Adriana Mika, Tomasz Sledzinski

**Affiliations:** 1 Department of Environmental Analysis, Faculty of Chemistry, University of Gdansk, Wita Stwosza 63, 80-308, Gdansk, Poland; 2 Physics-Chemistry Workshops, Faculty of Chemistry, University of Gdansk, Wita Stwosza 63, 80-308, Gdansk, Poland; 3 Department of General, Endocrine and Transplant Surgery, Medical University of Gdansk, Smoluchowskiego 17, 80-214, Gdansk, Poland; 4 Department of Pharmaceutical Biochemistry, Medical University of Gdansk, Debinki 1, 80-211, Gdansk, Poland

**Keywords:** Colorectal cancer, Triacylglycerols, Phospholipids, Sphingolipids, Liquid chromatography–mass spectrometry

## Abstract

**Background:**

Accumulating evidence indicates alterations in lipid metabolism and lipid composition in neoplastic tissue. Earlier nuclear magnetic resonance studies showed that the contents of major lipid groups, such as triacylglycerols, phospholipids and cholesterol, are changed in colon cancer tissue.

**Methods:**

In this study, a more detailed analysis of lipids in cancer and tumor adjacent tissues from colorectal cancer patients, using liquid chromatography–mass spectrometry, allowed for comparison of 199 different lipids between cancer tissue and tumor adjacent tissue using principal component analysis.

**Results:**

Significant differences were found in 67 lipid compounds between the two types of tissue; many of these lipid compounds are bioactive lipids such as ceramides, lysophospholipids or sterols and can influence the development of cancer. Additionally, increased levels of phospholipids and sphingolipids were present, which are major components of the cell membrane, and increases in these lipids can lead to changes in cell membrane properties.

**Conclusions:**

This study showed that many complex lipids are significantly increased or decreased in colon cancer tissue, reflecting significant alterations in lipid metabolism. This knowledge can be used for the selection of potential molecular targets of novel anticancer strategies based on the modulation of lipid metabolism and the composition of the cell membrane in colorectal cancer cells.

**Supplementary Information:**

The online version contains supplementary material available at 10.1186/s12944-021-01512-x.

## Background

Colorectal cancer (CRC), according to the 2020 GLOBOCAN statistics, is among the top three most frequently diagnosed types of cancer and most fatal cancers in people of both sexes worldwide [1]. Despite the favorable effects of early screening and appropriate surveillance in developed nations, disparities due to socioeconomic factors and an alarming increase in CRC diagnosis in patients under 50 years continue to make CRC a considerable global public health issue [1, 2]. Progress in decreasing the burden of CRC through improved prevention or treatment needs detailed insight into metabolic alterations associated with this malignancy.

Because of advances in sample preparation methods and analytical techniques [3], lipid analysis has emerged as a useful tool in cancer research [4–6]. In particular, the rapid development of mass spectrometry methods enables increasingly sensitive and precise analysis [7]. Dysregulation of lipid metabolism has emerged as one of the most prominent phenotypic hallmarks of cancer [5]. Lipids are a complex group of biomolecules with varying structures and functions, and their role in cellular processes cannot be overstated. They play a role in energy metabolism and membrane formation, are precursors for the synthesis of signaling molecules and are even involved in the regulation of gene expression via epigenetic modulations [4, 5, 8]. Changes in lipid composition in biofluids and tissues are persistently associated with CRC [6], and lipid metabolism is being explored as a potential therapeutic target for CRC [4, 9] and in biomarker discovery [10–13].

Despite this knowledge, evidence on the exact nature of lipid alterations in colorectal cancer tissues is unclear. Intracellular accumulation of lipid droplets (LDs), organelles rich in neutral lipids, mainly triacylglycerols (TGs) and cholesteryl esters (CEs), has been reported in a number of neoplastic processes, and LDs play a role in a number of cancer metabolic hallmarks, such as hypoxia, death evasion and cell proliferation and inflammation [14]. The demonstration that LDs are a major site of cyclooxygenase-2 (COX-2) activity and prostaglandin E2 (PGE2) production in CRC cells highlighted the functional significance of LDs in cancer, and a higher number of LDs was detected in CRC tumor tissue than in adjacent normal tissue [15]. Interestingly, investigation of CRC cell lines revealed that although differentiated tumor cells contain greater amounts of LDs than normal epithelial cell lines, the greatest amount of LDs was found in CRC stem cell lines [16]. Furthermore, the tumorigenic potential of cancer stem cells was linked to overaccumulation of LDs, strengthening the idea that LDs are important in carcinogenesis. Wu et al. described the association between CRC progression and overaccumulation of LDs in tumor-associated macrophages, which increase the ability of tumors to grow and metastasize [17]. Accumulation of LDs in CRC cells was also found to promote CRC chemoresistance [18]. Taken together, these results point to LD metabolism as a potential therapeutic target. However, despite reports of LD accumulation in CRC tissue/cells, CRC tissue seems to be characterized by a paradoxically lower content of TGs, an LD-associated lipid group, than normal, cancer-adjacent tissue [19–21]. In addition, in a previous study, the total lipid content in CRC tissues was lower than that in tumor adjacent healthy mucosa [21]. These disparities highlight the gaps in understanding the role of LDs in cancer and emphasize the need for further investigation.

Phospholipid (PL) analysis is also increasingly performed in cancer lipidomic studies. PLs are the basic components of cellular membranes and thereby affect many membrane-associated processes, e.g., regulation of homeostasis, cell adhesion and migration; cellular signaling; cell-cell interactions; vesicular trafficking; and apoptosis [6, 22]. Alterations in the composition and distribution of PLs in cells, tissues and biofluids are associated with cancer [22] and have been explored as potential diagnostic or prognostic biomarkers in a variety of cancers, such as breast cancer [23], prostate cancer [24], lung cancer [25] and ovarian cancer [26]. PL analysis has also been applied in CRC to investigate the suitability of model colon cell lines [27] and three-dimensional culture systems [13] for lipidomic analysis in CRC studies. Accumulation of PL species reflects the increased amounts of polyunsaturated fatty acids (PUFAs), a phenomenon that has previously been observed in CRC tissues [28]. PLs, whose change in abundance is associated with CRC, include lysophospholipids (LPLs), an important group of signaling lipids [12, 29], and ether lipids, which can function as endogenous antioxidants [29]. Characterization of PL profiles in CRC tissues by imaging enabled researchers to localize some phosphatidylcholine species in colorectal cancer tissue regions and establish differences between tumor-adjacent and tumor-remote tissues [30] and was also applied for in vivo CRC phenotyping [20]. Interestingly, matrix assisted laser desorption and ionization (MALDI) mass spectrometry imaging also revealed distinct PL signatures that are able to discriminate between six different types of cancer microenvironments, therefore suggesting the possibility of a distinct lipogenic mechanism involved in these malignant processes [31].

Altered lipid metabolism in cancer cells is a potential molecular target of anticancer therapy. The best-known strategies are directed toward the fatty acid (FA) synthesis pathway. The well-known strategy of inhibition of fatty acid synthase (FASN) and the efficacy of the FASN inhibitor TVB-2640 have been tested in phase I and II clinical trials [32]. Additionally, orlistat, an anti-obesity drug, which also inhibits FASN activity, has shown anticancer effects in CRC cells [33]. However, other lipids may also be molecular targets for anticancer therapy. Related strategies include decreasing the cholesterol content [34], modulating lipid domains in cell membranes [35], and targeting membrane fluidity by modulating the lipid composition [36]. Thus, a thorough understanding of lipid dysregulation in CRC is crucial. To this end, lipid composition between tumor adjacent colon mucosa and cancer tissue in CRC patients was analyzed by liquid chromatography–mass spectrometry (LC-MS) and compared.

## Methods

### Patients

This LC-MS study was conducted on tissue samples obtained during surgical resection from patients with CRC who were included in previous investigations of FA profiles by gas chromatography–mass spectrometry (GC-MS) [28] and lipid groups by nuclear magnetic resonance (NMR) [21] to obtain more accurate data on abnormalities in complex lipids in tumor tissue. In the present study, 10 patients were included with T2-T4 CRC according to the TNM classification with a mean age of 68.4 ± 9.02 years and a mean body mass index (BMI) of 29.0 ± 4.27, characteristics of each patient are included in Supplementary Table 1. The included patients underwent primary resection of the large bowel without neoadjuvant chemo- or radiotherapy. The samples were collected from the tumor and tumor adjacent, micro and macroscopically normal large intestinal mucosa within the resection margin immediately after surgical resection. Each sample was divided into two parts. Recently, a representative photographs of H&E-stained tumor adjacent and cancer tissues from this group of patients were published [21]. The part of the sample designated for the lipidomic study was frozen in liquid nitrogen immediately after collection and stored at − 80 °C until analysis. The other part was examined histopathologically to confirm or exclude cancer tissue.

### Lipids extraction

Samples were prepared as follows: aliquots of 50 mg of tumor and tumor adjacent tissue were homogenized in a chloroform-methanol mixture (1:1, v/v), saline was added, and the organic phase was collected and dried under a nitrogen stream. Prior to analysis, lipids were reconstituted in isopropanol to a final concentration of 1 mg/ml and passed through 0.2-μm PET filters.

### Analysis by liquid chromatography–mass spectrometry (LC-MS)

The procedure for LC-MS analysis followed a method modified from Ulmer et al. [37]. The high-performance LC-MS system employed consisted of an HCT Ultra spectrometer (Bruker Daltonics, Billerica, Massachusetts, US) with an ESI source coupled with an Agilent 1200 liquid chromatograph (Agilent Technologies, Santa Clara, California, US). Chromatographic separation was conducted on a ReproSil-Pur Basic-C18 column (5 μm, 150 × 4.6 mm; Dr. Maisch GmbH, Ammerbuch, Germany). Phase A consisted of 10 mM ammonium formate in acetonitrile-water (60:40, v/v) with 0.1% formic acid, and phase B consisted of 10 mM ammonium formate in isopropanol-acetonitrile-water (90:8:2, v/v/v) with 0.1% formic acid. The employed gradient was as follows: 0 min – 45% B, 15 min – 75% B, 20 min – 95% B, 30 min – 95% B, 33 min – 45% B, and 38 min – 45% B. The flow was set at 0.4 ml/min, and the injection volume was 10 μl. Spectra were acquired in positive ESI mode, the capillary voltage was set at 136 V, the scanned mass range was 50–1500 m/z, and the accumulation time was 200,000 ms. Representative total ion chromatograms of tumor and tumor adjacent tissues are shown in Supplementary Fig. 1. The HPLC-MS method was tested using a mixture of standards consisting of TG (18:1/18:1/18:1), TG (16:0/16:0/16:0), cholesterol, ceramides (Cers) Cer(d18:1/6:0), Cer(d18:1/18:1(9Z)), sphingosine-1-phosphate, phosphatidylcholine (PC) (18:1/16:0) and palmitic acid (purchased from Sigma Aldrich, St. Louis, MO, USA) in switched polarity mode to determine retention time windows for lipid groups.

### Data processing and statistical analysis

All spectra used for lipid identification were imported into SimLipid® 6.03 (PREMIER Biosoft, San Francisco, CA, USA) and preprocessed to exclude regions from 0 to 2.5 min and 28–38 min (column equilibration time). Lipid identification was done using high throughput MS lipid search, performed in the positive ion mode for [M + H], [M + NH4] ions, m/z tolerance 0.5 Da, in three time windows: 2–8 min, phosphatidylcholines (PC), phosphatidylethanolamines (PE), phosphatidylglycerols (PG), phosphatidylserine (PS); 8–23 min, glycerophospholipids, glycerolipids, sphingolipids, sterols; 22–28 min, glycerolipids, sterols, and results were reviewed manually. The m/z of observed adducts are presented in Supplementary Table 2. A peaklist alignment was performed, with maximum retention drift time across the runs 0.25 min, m/z tolerance of 0.05 and RT error tolerance set to the value of 0.2, peaks observed in < 50% of the samples were excluded. The data were normalized using the total response sum, log transformed and were subjected to Pareto scaling. Multivariate analysis and statistical data analysis were performed on a set of 199 unique assigned lipids in SIMCA software (version 16 Sartorius Stedim Data Analytics AB, Umeå, Sweden), only lipids that were present in at least 50% of the samples (both tumor and tumor adjacent tissues) were included in the analysis. The graphical representation of the results as a principal component analysis (PCA) biplot was constructed from the first two components, the software performs PCA model cross validation using the approach described by Eastment & Krzanowski [38]. Univariate analysis was performed with paired, two-tailed Student’s t-test.

## Results

**Fig. 1:**
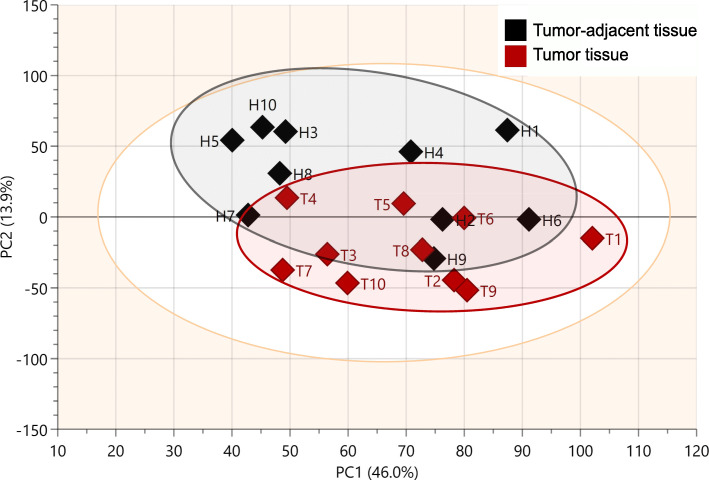
PCA plot for all analyzed lipid species

The method used allowed the detection of 199 different lipid species. PCA of the whole set of detected lipids showed that the lipid profile was different between tumor adjacent and CRC tissues (Fig. 1), although some overlapping was present.

**Fig. 2:**
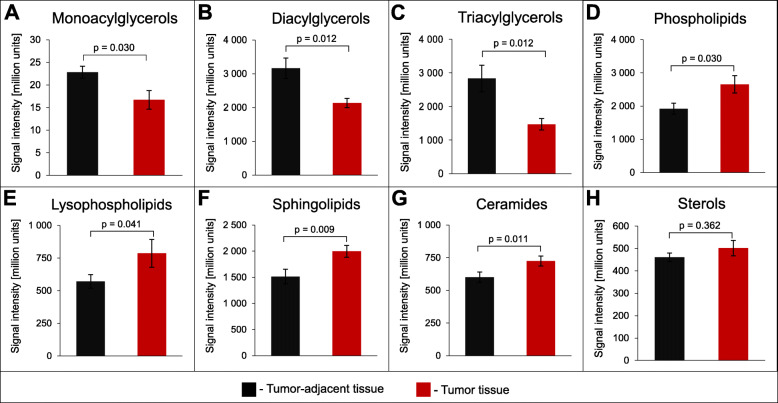
The sum of the signal intensities of the detected compounds within various groups of complex lipids. Values are mean ± SEM. *P*-value from paired, two-tailed t-Student’s test

Then, the sum of the signal intensities of the detected compounds within various groups of complex lipids between tumor adjacent mucosa and cancer tissue was compared. Among acylglycerols, the amounts of monoacylglycerols (MGs), diacylglycerols (DGs), and TGs were lower in tumor tissue than in tumor adjacent tissue (Fig. 2A-C). By contrast, the amounts of lipids forming cell membranes - PLs, LPLs, Cers and sphingolipids (SPLs) - were higher in tumor tissue (Fig. 2D-G). Only the amounts of sterols were similar between tumor tissue and tumor adjacent mucosa, with a slight increasing trend in tumor tissue (Fig. 2H).

The results for individual species that were significantly different between tumor and tumor adjacent tissues are presented in Table 1, whereas the results for the whole set of detected lipids are presented in Supplementary Table 2. Only one MG (18:1) was detected that was significantly lower in tumor tissue. Among the 29 detected DGs, 11 were significantly different between tumor and tumor adjacent tissue: 8 were lower and 3 were higher in tumor tissue. Among the 37 detected TGs, 21 were significantly higher in tumor tissue, and none were significantly higher in tumor adjacent tissue. There was no significant correlation between the *p* values of the differences and the length or degree of saturation of the FAs forming acylglycerols. Among the 53 detected PLs, the signals of 17 were significantly higher and none were lower in tumor tissue than in tumor adjacent tissue. The phospholipids whose amounts were significantly higher in tumor tissue included 7 PCs, 3 PEs, 2 PSs and one PG. Additionally, ether phospholipids, namely, one 1-(1Z-alkenyl),2-acylglycerophosphate, one 1-alkyl,2-acylglycerophosphate and two 1-alkyl,2-acylglycerophosphocholines, were more abundant in tumor tissue. 30 LPLs were identified, and among them, 6 were significantly more abundant in tumor tissue: one lysophosphatidylcholine (LPC), 2 lysophosphatidylethanolamines (LPEs), 2 lysophosphatidylglycerols (LPGs) and 1 lysophosphatidylserine (LPS). Among identified 34 SPLs, 8 were significantly higher in tumor tissue than in tumor adjacent colon mucosa: 4 Cers, 2 sphingomyelins (SMs) and 2 sphingosines. Finally, 15 sterols were detected, including 8 CEs, but unfortunately, this method was not able to detect free cholesterol, which is a main component of the cell membrane. Among the detected sterols, the amounts of three were significantly different in tumor tissue compared to tumor adjacent tissue. The amounts of 22:3 cholesteryl ester and 3,5-cholestadien-7-one were higher, whereas the signal of 24,25-epoxy-cholesterol was lower, in tumor tissue (Table 1).

**Table 1:** Species of lipids significantly different between tumor and tumor adjacent tissues of CRC patients

Name	Lipid class	Sub class	Tumor adjacent tissue	Tumor tissue	P
**Monoacylglycerols**					
MG(18:1)	MG	Monoacylglycerols	22.8 ± 1.34	16.7 ± 2.05	0.030
**Diacylglycerols**					
DG(29:2)	DG	Diacylglycerols	119 ± 20.0	61.9 ± 6.27	0.014
DG(32:0)	DG	Diacylglycerols	38.5 ± 5.72	22.1 ± 3.63	0.021
DG(33:1)	DG	Diacylglycerols	20.4 ± 2.47	32.5 ± 3.04	0.005
DG(33:3)	DG	Diacylglycerols	782 ± 120	335 ± 55.2	0.008
DG(33:4)	DG	Diacylglycerols	379 ± 55.4	206 ± 29.3	0.019
DG(33:5)	DG	Diacylglycerols	28.0 ± 5.96	12.7 ± 2.51	0.022
DG(35:4)	DG	Diacylglycerols	438 ± 68.7	237 ± 16.6	0.010
DG(35:5)	DG	Diacylglycerols	609 ± 93.7	294 ± 35.7	0.014
DG(35:6)	DG	Diacylglycerols	15.2 ± 1.49	8.62 ± 0.75	0.002
DG(36:0)	DG	Diacylglycerols	82.3 ± 18.6	169 ± 20.7	0.006
DG(44:5)	DG	Diacylglycerols	29.0 ± 4.43	51.5 ± 6.43	0.041
**Triacylglycerols**					
TG(44:2)	TG	Triacylglycerols	86.1 ± 25.1	32.8 ± 7.92	0.033
TG(43:1)	TG	Triacylglycerols	15.2 ± 1.80	7.73 ± 0.94	0.002
TG(44:0)	TG	Triacylglycerols	30.3 ± 4.80	15.8 ± 2.51	0.023
TG(46:1)	TG	Triacylglycerols	102 ± 20.4	26.9 ± 3.42	0.008
TG(46:2)	TG	Triacylglycerols	21.6 ± 4.08	9.76 ± 1.18	0.019
TG(47:1)	TG	Triacylglycerols	14.3 ± 1.93	6.79 ± 0.95	0.004
TG(47:2)	TG	Triacylglycerols	63.7 ± 14.7	27.7 ± 5.84	0.036
TG(47:4)	TG	Triacylglycerols	12.4 ± 1.63	9.11 ± 0.54	0.048
TG(47:6)	TG	Triacylglycerols	51.8 ± 9.95	28.0 ± 4.41	0.033
TG(48:1)	TG	Triacylglycerols	27.0 ± 4.70	13.1 ± 1.62	0.033
TG(48:2)	TG	Triacylglycerols	44.0 ± 7.78	19.5 ± 3.97	0.028
TG(48:3)	TG	Triacylglycerols	170 ± 29.9	71.0 ± 14.9	0.009
TG(48:4)	TG	Triacylglycerols	16.3 ± 1.24	10.0 ± 1.10	0.001
TG(50:1)	TG	Triacylglycerols	83.2 ± 12.2	39.8 ± 5.81	0.009
TG(50:3)	TG	Triacylglycerols	45.4 ± 6.63	21.7 ± 4.13	0.005
TG(50:4)	TG	Triacylglycerols	27.3 ± 4.56	14.5 ± 2.32	0.040
TG(52:2)	TG	Triacylglycerols	127 ± 20.0	74.9 ± 9.45	0.044
TG(52:3)	TG	Triacylglycerols	525 ± 102	283 ± 23.4	0.042
TG(52:4)	TG	Triacylglycerols	403 ± 49.6	177 ± 32.3	0.004
TG(52:5)	TG	Triacylglycerols	223 ± 39.2	98.8 ± 27.8	0.013
TG(53:1)	TG	Triacylglycerols	242 ± 41.4	127 ± 26.2	0.040
**Phospholipids**					
PC(26:0)	PC	Diacylglycerophosphocholines	11.2 ± 2.05	25.9 ± 6.86	0.043
PC(28:1)	PC	Diacylglycerophosphocholines	11.5 ± 2.35	38.4 ± 7.76	0.004
PC(30:0)	PC	Diacylglycerophosphocholines	10.0 ± 2.38	37.1 ± 7.85	0.004
PC(31:2)	PC	Diacylglycerophosphocholines	22.3 ± 2.26	30.5 ± 3.36	0.043
PC(31:4)	PC	Diacylglycerophosphocholines	0.89 ± 0.21	4.87 ± 1.27	0.010
PC(32:1)	PC	Diacylglycerophosphocholines	43.2 ± 20.2	79.3 ± 22.8	0.040
PC(34:1)	PC	Diacylglycerophosphocholines	125 ± 45.1	272 ± 69.5	0.038
PE(28:1)	PE	Diacylglycerophosphoethanolamines	5.99 ± 1.28	18.7 ± 5.77	0.038
PE(31:4)	PE	Diacylglycerophosphoethanolamines	1.72 ± 0.76	7.74 ± 2.64	0.033
PE(33:4)	PE	Diacylglycerophosphoethanolamines	9.47 ± 2.05	40.7 ± 8.60	0.003
PG(37:6)	PG	Diacylglycerophosphoglycerols	1.31 ± 0.47	4.66 ± 1.72	0.032
PS(16:0)	PS	Diacylglycerophosphoserines	9.27 ± 1.94	21.5 ± 4.89	0.037
PS(28:2)	PS	Diacylglycerophosphoserines	9.83 ± 2.02	35.7 ± 8.67	0.011
PA(P-39:1)	PA	1-(1Z-alkenyl),2-acylglycerophosphates	30.8 ± 2.43	43.4 ± 5.33	0.044
PA(O-38:1)	PA	1-alkyl,2-acylglycerophosphates	64.3 ± 11.3	141 ± 26.6	0.008
PC(O-36:4)	PC	1-alkyl,2-acylglycerophosphocholines	78.4 ± 13.8	165 ± 23.1	0.010
PC(O-37:2)	PC	1-alkyl,2-acylglycerophosphocholines	78.3 ± 12.3	99.7 ± 10.3	0.034
**Lysophospholipids**					
LPC(24:0)	PC	Monoacylglycerophosphocholines	10.8 ± 2.43	27.0 ± 6.86	0.023
LPE(16:1)	PE	Monoacylglycerophosphoethanolamines	5.66 ± 1.21	19.0 ± 4.69	0.013
LPE(18:2)	PE	Monoacylglycerophosphoethanolamines	23.3 ± 3.82	35.8 ± 4.55	0.036
LPG(20:4)	PG	Monoacylglycerophosphoglycerols	9.24 ± 1.95	22.2 ± 5.04	0.031
LPG(21:0)	PG	Monoacylglycerophosphoglycerols	6.52 ± 1.18	18.1 ± 5.11	0.045
LPS(O-20:0)	PS	Monoalkylglycerophosphoserines	40.3 ± 12.8	120 ± 31.0	0.037
**Sphingolipids**					
Cer(34:0)	Ceramides	N-acylsphinganines (dihydroceramides)	88.3 ± 20.8	128 ± 14.3	0.041
Cer(37:2)	Ceramides	N-acylsphingosines (ceramides)	18.7 ± 0.81	28.7 ± 3.92	0.043
Cer(38:1)	Ceramides	N-acylsphingosines (ceramides)	23.9 ± 1.97	37.5 ± 4.21	0.042
Cer(40:0)	Ceramides	N-acylsphinganines (dihydroceramides)	26.2 ± 3.35	38.6 ± 5.27	0.030
SM(34:2)	Phosphosphingolipids	Ceramide phosphocholines (sphingomyelins)	71.8 ± 14.3	97.4 ± 8.37	0.047
SM(39:2)	Phosphosphingolipids	Ceramide phosphocholines (sphingomyelins)	146 ± 22.8	211 ± 10.0	0.041
Sphingosine(14:2)	Sphingoid bases	Sphingoid base analogs	6.85 ± 0.74	9.67 ± 1.13	0.033
Sphingosine(18:3)	Sphingoid bases	Sphingoid base analogs	131 ± 34.6	245 ± 30.3	0.011
**Sterols**					
22:3 Cholesteryl ester	Sterols	Steryl esters	21.0 ± 3.33	35.9 ± 2.03	0.001
3,5-cholestadien-7-one	Sterols	Cholesterol and derivatives	18.3 ± 0.62	24.3 ± 2.20	0.038
24,25-epoxy-cholesterol	Sterols	Cholesterol and derivatives	19.4 ± 1.32	12.2 ± 1.47	0.001

Values are mean signal intensity (million units) ± SEM. P-value from paired, two-tailed t-Student’s test

## Discussion

This study showed that the lipidome of CRC tissue is different from that of tumor adjacent mucosa; however, not all complex lipids exhibited significant differences between these two types of tissue in the studied group of patients. The PCA model revealed that tumor adjacent and tumor tissue cluster separately, although the separation between these two groups was not complete. This can possibly be attributed to heterogeneity of tissues of each patient, since two components of the PCA model account for 59.9% of total variance (Fig. 1). This tendency to separate based on lipid profiles contrasts the results of a recent study by Wang et al. [29] who did not observe separation of normal mucosa and tumor based on two component PCA (39.45% of total variance). Among the 199 identified lipids, the amounts of 67 were significantly different between tumor adjacent and cancer tissue. The results suggest that the direction of changes depends on the role played by individual groups of lipids in CRC cells. Acylglycerols constitute an energy depot in lipid droplets that is used for energy generation in these metabolically active cells, whereas PLs, SPLs and free cholesterol are cell membrane components that are urgently needed during CRC cell proliferation. Comparison of these respective groups of lipids showed that in cancer cells, the levels of energy-providing acylglycerols are lower, whereas the levels of membrane-building lipids are higher. These results are in agreement with a previous ^1^H-NMR study, which also showed decreased TG and increased PL, SM and free cholesterol contents in tumor tissue compared to tumor adjacent colon mucosa [21]. However, the present study provided much more detailed data on specific lipids; moreover, additional groups of lipids, including MG and DGs, LPLs and Cers, were detected.

Thus far, the reports on the role of abundance of neutral lipids in CRC seem inconclusive. The elevated content of neutral lipid bearing LDs was previously associated with CRC [15, 16, 39]. While some studies indicate that the TG levels in cancerous tissue were significantly lower than in paracancerous/tumor adjacent tissue of CRC patients [19, 21, 40], another found no significant differences in TG content and LDs abundance and distribution [29]. Moreover, the reports differ with regards to CRC advancement and TG levels, with one study reporting higher levels of TGs in early stage tumors [19], and another enrichment of TG in T3 tumors [40]. Hama et al. described significant decrease of TGs with long-chain FA moieties, which form a majority of TG fraction, while simultaneously reported the elevation of TGs containing very-long chain FAs [41]. Here, the sum levels of triglycerides were significantly lower in tumor tissues. MG and DG are both precursors of TG synthesis by acyltransferases and products of TG hydrolysis by lipases. All these acylglycerols are finally hydrolyzed to produce glycerol and FAs, which, after activation by acyl-CoA synthetase and transport to mitochondria with the participation of the carnitine palmitoyltransferase 1 (CPT1), can be used for energy production by beta-oxidation to acetyl-CoA, which is then oxidized to CO_2_ in the Krebs cycle. A previous study showed increased expression of CPT1 in cancer tissue [21], which suggests that oxidation of FAs stored as TG in lipid droplets is a possible reason for the decrease in TG. The simultaneous decrease in MG and DG shown in this study supports this hypothesis. The lack of correlation between the *p* values of the differences and the length or degree of saturation of FAs forming acylglycerols suggests that there is no preference regarding the length or saturation of FAs during TG hydrolysis. However, the increased levels of PUFAs and saturated FAs (SFAs) and decreased levels of monounsaturated FAs (MUFAs) in CRC cells [28] suggest that SFAs and PUFAs are directed toward cell membrane synthesis, whereas MUFAs are preferentially used as an energy source and undergo beta-oxidation. It is also worth mentioning that MUFAs, which can be synthesized by stearoyl-CoA desaturase-1 (SCD1) in human cells [42], are the main component of TG and that SCD1 is overexpressed in many types of cancer, including CRC [6, 43–45]. It seems that overexpression of both SCD1 and FASN [33] is associated with increased production of TG, which include MUFAs that are conversely largely oxidized to provide energy to cancer cells. Another source of FAs for beta-oxidation may be the import of free FAs that are released from adipocytes adjacent to cancer cells [46]. DG is also a signaling molecule associated with the development of various cancers, but its role is associated with the activity of diacylglycerol kinases that target DG originating from hydrolysis of cell membrane PLs by phospholipase C [47], whereas the pool of DG in lipid droplets seems to be associated with the synthesis/degradation of TG in cancer cells. Recently, the issue of concurrent fatty acid synthesis and oxidation was extensively discussed in a review published by De Olivera et al. [48]. This group proposed the existence of two types of mitochondria—lipogenic mitochondria, which release citrate into the cytosol to fuel lipogenesis, and fatty acid oxidizing mitochondria, which produce ATP from fat [48].

In addition, PLs, LPLs and SPLs are cell membrane components, and their levels were increased in tumor tissue. This phenomenon is probably associated with increased synthesis of plasma membranes in rapidly proliferating cancer cells, as was suggested in a previous study [21]. Guo et al. [31], using MALDI-MSI, studied various cancers, including samples from six patients with CRC. Their results differed from those of described in this study; they found increases in MUFAs and decreases in PUFAs, as well as increases in PC containing MUFAs, but decreases in PC, phosphatidylinositol (PI) and PE containing PUFAs in cancer tissue. Surprisingly, they suggested that the synthesis of PUFAs from 18:1 FAs is downregulated [31], whereas this process is not possible in human tissues due to the lack of delta-12 and delta-15 desaturases [49]. By contrast, in this study, all significantly different PLs, both those containing MUFAs and those containing PUFAs, were more abundant in cancer tissue. This phenomenon may be associated with increased lipogenesis, lipolysis and exogenous FA intake—processes that provide FAs for the synthesis of membrane phospholipids and other membrane lipids [50]. The last process is especially important in the case of exogenous PUFAs (18:2 n-6 and 18:3 n-3) [28], which cannot be synthesized in human cells [49]. Increased levels of ether phospholipids were also detected. Ether lipids are increased in cancers and correlate with greater aggressiveness [51]. However, their exact role in promoting cancer progression is not known. It has been shown that they regulate ion channels, which may constitute the mechanism underlying the regulation of cell proliferation [52]. Likewise, the exact nature of sphingolipid metabolism in carcinogenic processes is not yet clear, but seems to be dependent on acyl-chain composition [53]. In current study, the difference in total Cer amounts between tumor and tumor adjacent tissue (Fig. 1) are in agreement with previous reports of elevated Cer synthases expression levels [54], as well as total Cer content in CRC tissues [29, 53].

The overall LPL content, in which LPCs are major contributors, detected here was increased, which contrasts with the results of Wang et al. [29], wherein they reported significant decrease across LPL classes (LPC, LPE) and increase in lysophosphatidylinositol (LPI). Contrary to that, Kitamura et al. [55] reported significantly higher levels of LPI and LPS in colon cancer tissue, and higher, although not statistically significant, levels of LPC, LPE and LPG. The results of present study support Kitamura et al. findings, the direction of change of particular species: LPE(16:1), LPE(18:2), LPG(20:4) (Table 1), aligns with their results, albeit the upregulation of these species in Kitamura et al. study lacked statistical significance, which may possibly arise due to small number of patients in both studies. The upregulation of LPLs seems surprising, considering that overexpression of enzymes LPCAT1 and LPCAT2, which re-acylate LPCs into PCs, was observed in CRC cells [18, 56]. However, these enzymes are associated with LDs, which exhibit high inter-individual variation that may possibly explain these opposing findings [29]. LPLs are precursors of lysophosphatidic acid (LPA), which is a signaling molecule and can inhibit p53 activity by activating the LPA receptor [57]. In addition, some ceramides activate p53 pathways [57]. Thus, both LPLs and ceramides may influence cancer cell proliferation and apoptosis; however, since both groups of lipids are increased in cancer tissue, it is hard to speculate about the combined effect of these compounds on p53 pathways. Additionally, LPE can act as a signaling molecule, stimulating the migration and invasion of human ovarian cancer cells by interacting with G protein-coupled receptors [58]. In addition, LPS is associated with inflammation [59], which is a characteristic factor for the development of CRC [6]. Moreover, their proinflammatory properties increase with the FA chain length [60], and in current study a significantly increased amount of LPS containing 20:0 FAs was present. SPLs are involved in the regulation of cell differentiation, proliferation and apoptosis, as well as drug resistance [61–63]; thus, increased levels of lipids from this group may also be important for CRC cell metabolism.

Among sterols, only 3 metabolites were significantly different between tumor and cancer tissues. One of them was 22:3 CE, which was increased in tumor tissue, but all seven other CEs did not differ significantly between tumor adjacent and tumor tissue, and the total sterol amount was not significantly different. The largely preserved CE profile is consistent with findings of Hama et al. [41], although it must be noted that in this study number of detected CE species was limited. CEs are strongly hydrophobic molecules and are located in lipid droplets. Unfortunately, the procedure used did not allow for detection of free cholesterol, which is an important component of cell membranes, by this method, but a previous study using ^1^H-NMR, showed that the level of free cholesterol is significantly higher in CRC tissue [21], which is consistent with the concept of the increased content of membrane lipids in CRC cells. Increased free cholesterol levels in cancer cell membranes are associated with increased levels of lipid rafts and increased resistance to anticancer drugs [46]. Another significant metabolite may contribute to increased levels of free cholesterol. 24(S),25-epoxycholesterol is a negative regulator of HMG-CoA reductase (HMGCR), the rate-limiting enzyme in cholesterol synthesis, that acts by binding to Liver X Receptor (LXR) [64]. A previous study showed increased expression of HMGCR in CRC tissue [21]. The present analysis revealed a significantly (approximately 40%) lower level of 24(S),25-epoxycholesterol in tumor tissue than in tumor adjacent tissue; thus, this decreased level may be one of the reasons for the elevated cholesterol synthesis and elevated free cholesterol levels in CRC cells. Interestingly, upregulation of 24(R/S),25-epoxycholesterol inhibits the proliferation of gastric cancer cells [65]. Another interesting sterol is 3,5 cholestadiene-7-one, which is a product of membrane cholesterol autooxidation [66], a process caused by oxidative stress that is present in cancer tissue [48].

### Study strengths and limitations

The most important advantage of this study was further discovering and confirming the altered lipid metabolism in CRC tissue. The results partly confirmed our previous studies [11, 21, 28, 67], but also provided a lot of new data. The advantage of this study was also using the matched tumor adjacent, micro and macroscopically normal colon mucosa and tumor samples from the same patients. Although the issue of field cancerization has been raised before [68] the paired tissue samples seem to be the most suitable controls, given the apparent heterogeneity of tissue samples [29, 53].

The limitations of the study include small number of samples, which did not allow to analyze correlations between demographic and clinical data and the results of lipidomic analysis. Also, the limited sensitivity of the LC-MS setup necessarily restrained lipid identification reporting up to lipid species/bond type level, due to the lack of MS/MS data. On the other hand, using this relatively simple approach, the trend for separation between tumor and tumor adjacent tissue could be observed and the ease of the procedure could be advantageous when expanding the cohort size.

## Conclusions

In conclusion, this study identified many complex lipids that are significantly increased or decreased in CRC tissue. These data extend the knowledge on alterations in the composition of CRC tissue. This knowledge can be used for the selection of potential molecular targets of novel anticancer strategies based on the modulation of lipid metabolism and the composition of the cell membrane in CRC cells. A detailed understanding of the observed alterations requires analyses of tissues from primary tumors stratified by the clinical stage of CRC. It seems probable that the lipid composition changes with the accumulation of mutations and progression from adenoma to carcinoma (in a well-described adenoma-to-carcinoma sequence) and further through the clinical stages of CRC. Defining alterations in locally advanced (stage I and II), regionally advanced (stage III) and disseminated (stage IV) disease would enable an understanding of the role of lipids in CRC progression. In future, this knowledge could also transfer into better CRC classification and possibly better inclusion criteria for adjuvant therapy if significant association between lipid profiles and CRC prognosis could be established. Moreover, it would be of interest to define alterations in individual cancer grades (from grade 1, where cancer cells look similar to tumor adjacent mucosal cells (well differentiated) to grade 3, where cancer cells look very abnormal (poorly differentiated).

## Supplementary Information

**Additional file 1: Figure S1.** Total ion chromatograms of representative samples from tumor adjacent (**A**) and tumor (**B**) tissues. Abbreviations: CE, cholesteryl ester; Cer, ceramide; DG, diacylglycerol; LPL, lysophospholipids; MG, monoacylglycerol; PA, phosphatidic acid; PC, phosphatidylcholine; PE, phosphatidylethanolamine; PG, phosphatidylglycerol; PHL, phospholipid; PS, phosphatidylserine; SM, sphingomyelin; TG, triacylglycerol.

**Additional file 2: Table S1.** Characteristics of colorectal cancer patients included in the study. Abbreviations: BPH, benign prostatic hyperplasia; COPD; chronic obstructive pulmonary disease; HT, hypertension; RA, rheumatoid arthritis; T2DM, type 2 diabetes.

**Additional file 3: Table S2.** Species of lipids detected in tumor and tumor adjacent tissues of CRC patients. Values are mean signal intensity (million units) ± SEM. *P*-value from paired, two-tailed t-Student’s test.

## Data Availability

The dataset analyzed in this study can be reasonably obtained from the corresponding author.

## Abbreviations

BMI: body mass index
CE: cholesteryl ester
Cer: ceramide
COX: cyclooxygenase
CPT1: carnitine palmitoyltransferase 1
CRC: colorectal cancer
DG: diacylglycerol
FA: fatty acid
FASN: fatty acid synthase
GC-MS: gas chromatography–mass spectrometry
HMGCR: HMG-CoA reductase
LC-MS: liquid chromatography–mass spectrometry
LD: lipid droplet
LPC: lysophosphatidylcholine
LPE: lysophosphatidylethanolamine
LPG: lysophosphatidylglycerol
LPI: lysophosphatidylinositol
LPL: lysophospholipid
LPS: lysophosphatidylserine
LXR: Liver X Receptor
MG: monoacylglycerol
MALDI: matrix assisted laser desorption and ionization
MUFA: monounsaturated fatty acid
NMR: nuclear magnetic resonance
PC: phosphatidylcholine
PCA: principal component analysis
PE: phosphatidylethanolamine
PG: phosphatidylglycerol
PGE2: prostaglandin E2
PI: phosphatidylinositol
PL: phospholipid
PS: phosphatidylserine
PUFA: polyunsaturated fatty acid
SCD1: stearoyl-CoA desaturase-1
SFA: saturated fatty acid
SM: sphingomyelin
SPL: sphingolipid
TAG: triacylglycerol
