# Supplementary material for: Alterations in complex lipids in tumor tissue of patients with colorectal cancer
Source: Lipids Health Dis. 2021 Aug 4;20:85. doi: 10.1186/s12944-021-01512-x (PMC8340484; doi:10.1186/s12944-021-01512-x)
Supplement: Supplementary file 1 — Additional file 1: Figure S1. Total ion chromatograms of representative samples from tumor adjacent (A) and tumor (B) tissues. Abbreviations: CE, cholesteryl ester; Cer, ceramide; DG, diacylglycerol; LPL, lysophospholipids; MG, monoacylglycerol; PA, phosphatidic acid; PC, phosphatidylcholine; PE, phosphatidylethanolamine; PG, phosphatidylglycerol; PHL, phospholipid; PS, phosphatidylserine; SM, sphingomyelin; TG, triacylglycerol. [file 12944_2021_1512_MOESM1_ESM.docx]

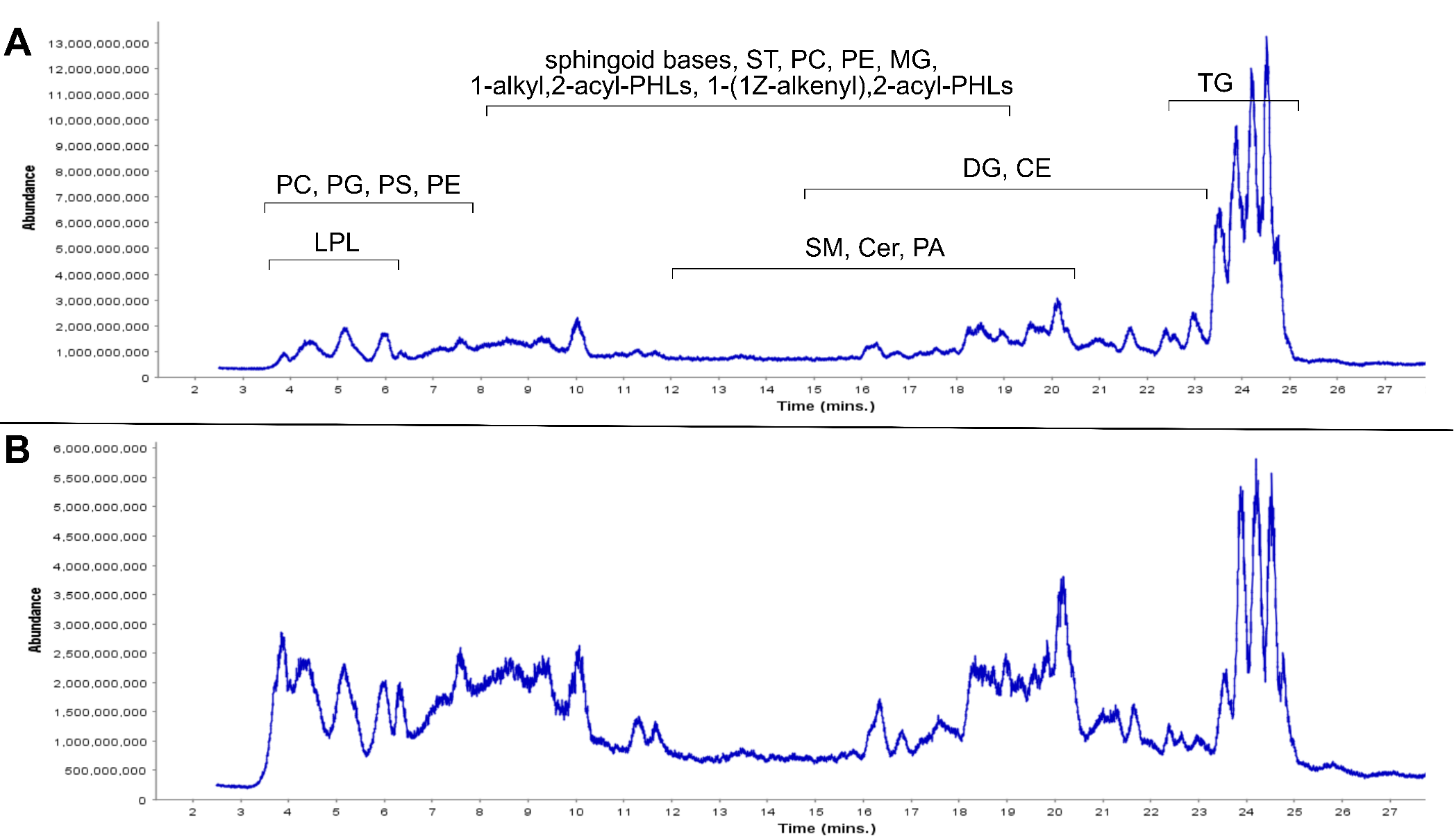


**Supplementary Figure 1. Total ion chromatograms of representative samples from tumor adjacent (A) and tumor (B) tissues.** Abbreviations: CE, cholesteryl ester; Cer, ceramide; DG, diacylglycerol; LPL, lysophospholipids; MG, monoacylglycerol; PA, phosphatidic acid; PC, phosphatidylcholine; PE, phosphatidylethanolamine; PG, phosphatidylglycerol; PHL, phospholipid; PS, phosphatidylserine; SM, sphingomyelin; TG, triacylglycerol.
