# Supplementary material for: Alterations in complex lipids in tumor tissue of patients with colorectal cancer
Source: Lipids Health Dis. 2021 Aug 4;20:85. doi: 10.1186/s12944-021-01512-x (PMC8340484; doi:10.1186/s12944-021-01512-x)
Supplement: Supplementary file 2 — Additional file 2: Table S1. Characteristics of colorectal cancer patients included in the study. Abbreviations: BPH, benign prostatic hyperplasia; COPD; chronic obstructive pulmonary disease; HT, hypertension; RA, rheumatoid arthritis; T2DM, type 2 diabetes. [file 12944_2021_1512_MOESM2_ESM.docx]

**Supplementary Table 1. Characteristics of colorectal cancer patients included in the study.**

| **Patient** | **Sex** | **Age** | **BMI** | **Location of primary tumor** | **T stage** | **Lymph nodes** | **Metastases** | **Grade** | **Statins** | **Comorbidities** |
| --- | --- | --- | --- | --- | --- | --- | --- | --- | --- | --- |
| CRC1 | male | 66 | 38 | Sigmoid colon | 4a | 2b | 0 | 2 | none | HT, T2DM |
| CRC2 | male | 71 | 27 | Rectosigmoid junction | 3 | 1b | 0 | 2 | none | HT, asthma,  post laryngeal cancer |
| CRC3 | male | 73 | 29 | Transverse colon | 4b | 1a | 0 | 2 | atorvastatin | HT, BPH |
| CRC4 | male | 57 | 26 | Sigmoid colon | 2 | 0 | 0 | 2 | none | none |
| CRC5 | male | 51 | 33 | Rectum | 3 | 1b | 0 | 2 | none | HT |
| CRC6 | male | 75 | 29 | Rectosigmoid junction | 3 | 1c | 0 | 2 | none | COPD |
| CRC7 | male | 64 | 30 | Rectosigmoid junction | 3 | 1b | 0 | 2 | none | HT, BPH, hyperthyroidism |
| CRC8 | female | 81 | 25 | Transverse colon | 3 | 0 | 0 | NA | rosuvastatin | HT, RA, T2DM, hypothyroidism, hypercholesterolemia |
| CRC9 | male | 74 | 23 | Rectosigmoid junction | 3 | 1a | 0 | 3 | rosuvastatin | HT, BPH |
| CRC10 | female | 72 | 30 | Transverse colon | 3 | 0 | 0 | 3 | none | HT, asthma |

Abbreviations: BPH, benign prostatic hyperplasia; COPD; chronic obstructive pulmonary disease; HT, hypertension; RA, rheumatoid arthritis; T2DM, type 2 diabetes.
