# Supplementary material for: Alterations in complex lipids in tumor tissue of patients with colorectal cancer
Source: Lipids Health Dis. 2021 Aug 4;20:85. doi: 10.1186/s12944-021-01512-x (PMC8340484; doi:10.1186/s12944-021-01512-x)
Supplement: Supplementary file 3 — Additional file 3: Table S2. Species of lipids detected in tumor and tumor adjacent tissues of CRC patients. Values are mean signal intensity (million units) ± SEM. P-value from paired, two-tailed t-Student’s test. [file 12944_2021_1512_MOESM3_ESM.docx]

**Supplementary Table 2. Species of lipids detected in tumor and tumor adjacent tissues of CRC patients.**

| **Name** | **m/z** | **Adduct** | **Lipid class** | **Sub class** | **Tumor adjacent tissue** | **Tumor tissue** | **P** |
| --- | --- | --- | --- | --- | --- | --- | --- |
| **Monoacylglycerols** |  |  |  |  |  |  |  |
| MG(18:1) | 374.6 | [M+NH4]+ | MG | Monoacylglycerols | 22.8 ± 1.34 | 16.7 ± 2.05 | 0.030 |
| **Diacylglycerols** |  |  |  |  |  |  |  |
| DG(28:0) | 518.6 | [M+H]+ | DG | Diacylglycerols | 27.0 ± 3.58 | 25.4 ± 5.81 | 0.858 |
| DG(29:2) | 523.4 | [M+H]+ | DG | Diacylglycerols | 119 ± 20.0 | 61.9 ± 6.27 | 0.014 |
| DG(30:0) | 558.6 | [M+NH4]+ | DG | Diacylglycerols | 1.48 ± 0.13 | 1.93 ± 0.34 | 0.186 |
| DG(30:2) | 537.6 | [M+H]+ | DG | Diacylglycerols | 42.0 ± 8.21 | 53.4 ± 4.11 | 0.214 |
| DG(32:0) | 569.6 | [M+H]+ | DG | Diacylglycerols | 38.5 ± 5.72 | 22.1 ± 3.63 | 0.021 |
| DG(32:2) | 565.8 | [M+H]+ | DG | Diacylglycerols | 102 ± 21.4 | 103 ± 24.8 | 0.953 |
| DG(32:4) | 561.6 | [M+H]+ | DG | Diacylglycerols | 22.2 ± 1.65 | 21.9 ± 1.80 | 0.872 |
| DG(32:5) | 576.6 | [M+NH4]+ | DG | Diacylglycerols | 19.4 ± 1.94 | 19.8 ± 3.12 | 0.926 |
| DG(33:1) | 598.6 | [M+NH4]+ | DG | Diacylglycerols | 20.4 ± 2.47 | 32.5 ± 3.04 | 0.005 |
| DG(33:3) | 577.8 | [M+H]+ | DG | Diacylglycerols | 782 ± 120 | 335 ± 55.2 | 0.008 |
| DG(33:4) | 575.6 | [M+H]+ | DG | Diacylglycerols | 379 ± 55.4 | 206 ± 29.3 | 0.019 |
| DG(33:5) | 537.4 | [M+H]+ | DG | Diacylglycerols | 28.0 ± 5.96 | 12.7 ± 2.51 | 0.022 |
| DG(34:2) | 593.8 | [M+H]+ | DG | Diacylglycerols | 33.0 ± 5.19 | 42.9 ± 2.09 | 0.099 |
| DG(34:5) | 604.8 | [M+NH4]+ | DG | Diacylglycerols | 21.0 ± 2.06 | 25.2 ± 4.13 | 0.360 |
| DG(35:4) | 603.6 | [M+H]+ | DG | Diacylglycerols | 438 ± 68.7 | 237 ± 16.6 | 0.010 |
| DG(35:5) | 601.6 | [M+H]+ | DG | Diacylglycerols | 609 ± 93.7 | 294 ± 35.7 | 0.014 |
| DG(35:6) | 616.6 | [M+NH4]+ | DG | Diacylglycerols | 15.2 ± 1.49 | 8.62 ± 0.75 | 0.002 |
| DG(36:0) | 625.8 | [M+H]+ | DG | Diacylglycerols | 82.3 ± 18.6 | 169 ± 20.7 | 0.006 |
| DG(36:2) | 638.6 | [M+NH4]+ | DG | Diacylglycerols | 57.6 ± 4.42 | 49.7 ± 5.40 | 0.319 |
| DG(36:4) | 634.8 | [M+NH4]+ | DG | Diacylglycerols | 69.0 ± 8.39 | 88.9 ± 12.3 | 0.144 |
| DG(36:4) | 622.8 | [M+H]+ | DG | Diacylglycerols | 15.8 ± 1.14 | 11.9 ± 1.76 | 0.089 |
| DG(36:5) | 632.8 | [M+NH4]+ | DG | Diacylglycerols | 101 ± 13.4 | 125 ± 29.5 | 0.377 |
| DG(39:0) | 667.6 | [M+H]+ | DG | Diacylglycerols | 1.93 ± 0.20 | 1.69 ± 0.18 | 0.399 |
| DG(39:6) | 672.6 | [M+NH4]+ | DG | Diacylglycerols | 1.32 ± 0.23 | 1.92 ± 0.36 | 0.080 |
| DG(40:6) | 669.6 | [M+H]+ | DG | Diacylglycerols | 20.9 ± 1.42 | 21.4 ± 3.11 | 0.882 |
| DG(41:7) | 698.6 | [M+NH4]+ | DG | Diacylglycerols | 36.6 ± 5.66 | 51.2 ± 7.37 | 0.159 |
| DG(42:5) | 716.6 | [M+NH4]+ | DG | Diacylglycerols | 25.7 ± 3.51 | 36.9 ± 4.99 | 0.097 |
| DG(42:7) | 712.7 | [M+NH4]+ | DG | Diacylglycerols | 23.7 ± 4.34 | 23.2 ± 4.66 | 0.928 |
| DG(44:5) | 744.8 | [M+NH4]+ | DG | Diacylglycerols | 29.0 ± 4.43 | 51.5 ± 6.43 | 0.041 |
| **Triacylglycerols** |  |  |  |  |  |  |  |
| TG(38:0) | 684.6 | [M+NH4]+ | TG | Triacylglycerols | 21.0 ± 2.15 | 18.1 ± 1.31 | 0.240 |
| TG(42:4) | 732.4 | [M+NH4]+ | TG | Triacylglycerols | 1.95 ± 0.36 | 2.20 ± 0.52 | 0.643 |
| TG(43:1) | 752.5 | [M+NH4]+ | TG | Triacylglycerols | 15.2 ± 1.80 | 7.73 ± 0.94 | 0.002 |
| TG(44:0) | 796.8 | [M+NH4]+ | TG | Triacylglycerols | 30.3 ± 4.80 | 15.8 ± 2.51 | 0.023 |
| TG(44:1) | 766.8 | [M+NH4]+ | TG | Triacylglycerols | 10.8 ± 1.10 | 11.1 ± 0.74 | 0.817 |
| TG(44:2) | 764.8 | [M+NH4]+ | TG | Triacylglycerols | 86.1 ± 25.1 | 32.8 ± 7.92 | 0.033 |
| TG(44:3) | 762.8 | [M+NH4]+ | TG | Triacylglycerols | 2.17 ± 0.36 | 1.94 ± 0.37 | 0.697 |
| TG(45:2) | 778.6 | [M+NH4]+ | TG | Triacylglycerols | 1.50 ± 0.34 | 1.16 ± 0.10 | 0.424 |
| TG(45:5) | 772.8 | [M+NH4]+ | TG | Triacylglycerols | 10.0 ± 1.64 | 15.4 ± 3.46 | 0.151 |
| TG(46:1) | 794.8 | [M+NH4]+ | TG | Triacylglycerols | 102 ± 20.4 | 26.9 ± 3.42 | 0.008 |
| TG(46:2) | 792.8 | [M+NH4]+ | TG | Triacylglycerols | 21.6 ± 4.08 | 9.76 ± 1.18 | 0.019 |
| TG(46:4) | 788.8 | [M+NH4]+ | TG | Triacylglycerols | 21.7 ± 1.06 | 18.2 ± 2.34 | 0.292 |
| TG(47:1) | 808.7 | [M+NH4]+ | TG | Triacylglycerols | 14.3 ± 1.93 | 6.79 ± 0.95 | 0.004 |
| TG(47:2) | 806.8 | [M+NH4]+ | TG | Triacylglycerols | 63.7 ± 14.7 | 27.7 ± 5.84 | 0.036 |
| TG(47:3) | 787.8 | [M+H]+ | TG | Triacylglycerols | 19.3 ± 1.36 | 18.6 ± 3.07 | 0.835 |
| TG(47:4) | 802.6 | [M+NH4]+ | TG | Triacylglycerols | 12.4 ± 1.63 | 9.11 ± 0.54 | 0.048 |
| TG(47:6) | 798.6 | [M+NH4]+ | TG | Triacylglycerols | 51.8 ± 9.95 | 28.0 ± 4.41 | 0.033 |
| TG(48:1) | 822.8 | [M+NH4]+ | TG | Triacylglycerols | 27.0 ± 4.70 | 13.1 ± 1.62 | 0.033 |
| TG(48:2) | 820.8 | [M+NH4]+ | TG | Triacylglycerols | 44.0 ± 7.78 | 19.5 ± 3.97 | 0.028 |
| TG(48:3) | 818.8 | [M+NH4]+ | TG | Triacylglycerols | 170 ± 29.9 | 71.0 ± 14.9 | 0.009 |
| TG(48:4) | 816.8 | [M+NH4]+ | TG | Triacylglycerols | 16.3 ± 1.24 | 10.0 ± 1.10 | 0.001 |
| TG(48:6) | 795.6 | [M+H]+ | TG | Triacylglycerols | 76.9 ± 17.7 | 56.7 ± 9.85 | 0.309 |
| TG(50:1) | 850.8 | [M+NH4]+ | TG | Triacylglycerols | 83.2 ± 12.2 | 39.8 ± 5.81 | 0.009 |
| TG(50:2) | 848.8 | [M+NH4]+ | TG | Triacylglycerols | 43.2 ± 8.27 | 25.7 ± 2.25 | 0.052 |
| TG(50:3) | 846.8 | [M+NH4]+ | TG | Triacylglycerols | 45.4 ± 6.63 | 21.7 ± 4.13 | 0.005 |
| TG(50:4) | 844.8 | [M+NH4]+ | TG | Triacylglycerols | 27.3 ± 4.56 | 14.5 ± 2.32 | 0.040 |
| TG(51:2) | 862.8 | [M+NH4]+ | TG | Triacylglycerols | 1.32 ± 0.31 | 1.63 ± 0.23 | 0.267 |
| TG(52:1) | 878.8 | [M+NH4]+ | TG | Triacylglycerols | 30.6 ± 7.06 | 19.9 ± 4.66 | 0.258 |
| TG(52:2) | 876.8 | [M+NH4]+ | TG | Triacylglycerols | 127 ± 20.0 | 74.9 ± 9.45 | 0.044 |
| TG(52:3) | 874.8 | [M+NH4]+ | TG | Triacylglycerols | 525 ± 102 | 283 ± 23.4 | 0.042 |
| TG(52:4) | 872.8 | [M+NH4]+ | TG | Triacylglycerols | 403 ± 49.6 | 177 ± 32.3 | 0.004 |
| TG(52:5) | 870.8 | [M+NH4]+ | TG | Triacylglycerols | 223 ± 39.2 | 98.8 ± 27.8 | 0.013 |
| TG(53:1) | 893 | [M+NH4]+ | TG | Triacylglycerols | 242 ± 41.4 | 127 ± 26.2 | 0.040 |
| TG(53:5) | 867.8 | [M+H]+ | TG | Triacylglycerols | 1.21 ± 0.19 | 2.00 ± 0.36 | 0.069 |
| TG(54:2) | 904.8 | [M+NH4]+ | TG | Triacylglycerols | 19.5 ± 3.97 | 12.2 ± 2.10 | 0.116 |
| TG(54:7) | 894.8 | [M+NH4]+ | TG | Triacylglycerols | 11.5 ± 1.28 | 9.30 ± 0.51 | 0.154 |
| TG(55:8) | 906.8 | [M+NH4]+ | TG | Triacylglycerols | 233 ± 38.6 | 141 ± 31.8 | 0.100 |
| **Phospholipids** |  |  |  |  |  |  |  |
| PA(29:2) | 603.1 | [M+H]+ | PA | Diacylglycerophosphates | 18.3 ± 3.00 | 20.6 ± 1.85 | 0.619 |
| PA(38:0) | 750.6 | [M+NH4]+ | PA | Diacylglycerophosphates | 33.7 ± 3.10 | 45.4 ± 6.22 | 0.134 |
| PA(40:2) | 774.6 | [M+NH4]+ | PA | Diacylglycerophosphates | 76.6 ± 8.90 | 79.2 ± 11.9 | 0.811 |
| PA(41:3) | 786.6 | [M+NH4]+ | PA | Diacylglycerophosphates | 83.7 ± 13.0 | 104 ± 17.3 | 0.111 |
| PC(8:0) | 397.8 | [M+H]+ | PC | Diacylglycerophosphocholines | 21.8 ± 1.18 | 17.8 ± 1.94 | 0.139 |
| PC(14:0) | 482.0 | [M+H]+ | PC | Diacylglycerophosphocholines | 21.5 ± 2.49 | 21.7 ± 2.75 | 0.955 |
| PC(26:0) | 667.5 | [M+NH4]+ | PC | Diacylglycerophosphocholines | 11.2 ± 2.05 | 25.9 ± 6.86 | 0.043 |
| PC(28:1) | 693.6 | [M+NH4]+ | PC | Diacylglycerophosphocholines | 11.5 ± 2.35 | 38.4 ± 7.76 | 0.004 |
| PC(28:2) | 674.5 | [M+H]+ | PC | Diacylglycerophosphocholines | 23.9 ± 2.03 | 30.7 ± 5.02 | 0.157 |
| PC(30:0) | 706.6 | [M+H]+ | PC | Diacylglycerophosphocholines | 10.0 ± 2.38 | 37.1 ± 7.85 | 0.004 |
| PC(30:3) | 700.9 | [M+H]+ | PC | Diacylglycerophosphocholines | 18.3 ± 2.59 | 30.6 ± 5.22 | 0.126 |
| PC(31:2) | 733.6 | [M+NH4]+ | PC | Diacylglycerophosphocholines | 22.3 ± 2.26 | 30.5 ± 3.36 | 0.043 |
| PC(31:4) | 729.6 | [M+NH4]+ | PC | Diacylglycerophosphocholines | 0.89 ± 0.21 | 4.87 ± 1.27 | 0.010 |
| PC(32:1) | 732.6 | [M+H]+ | PC | Diacylglycerophosphocholines | 43.2 ± 20.2 | 79.3 ± 22.8 | 0.040 |
| PC(33:0) | 748.8 | [M+H]+ | PC | Diacylglycerophosphocholines | 1.63 ± 0.39 | 2.84 ± 0.68 | 0.122 |
| PC(33:2) | 761.6 | [M+NH4]+ | PC | Diacylglycerophosphocholines | 4.60 ± 1.27 | 10.6 ± 4.30 | 0.185 |
| PC(34:1) | 760.6 | [M+H]+ | PC | Diacylglycerophosphocholines | 125 ± 45.1 | 272 ± 69.5 | 0.038 |
| PC(34:2) | 758.6 | [M+H]+ | PC | Diacylglycerophosphocholines | 106 ± 20.0 | 104 ± 27.0 | 0.923 |
| PC(37:5) | 811.6 | [M+NH4]+ | PC | Diacylglycerophosphocholines | 1.50 ± 0.33 | 5.62 ± 1.84 | 0.055 |
| PE(28:1) | 634.6 | [M+H]+ | PE | Diacylglycerophosphoethanolamines | 5.99 ± 1.28 | 18.7 ± 5.77 | 0.038 |
| PE(30:2) | 660.9 | [M+H]+ | PE | Diacylglycerophosphoethanolamines | 5.72 ± 1.06 | 21.5 ± 7.82 | 0.066 |
| PE(31:2) | 674.4 | [M+H]+ | PE | Diacylglycerophosphoethanolamines | 9.54 ± 0.91 | 22.3 ± 6.50 | 0.082 |
| PE(31:4) | 670.2 | [M+H]+ | PE | Diacylglycerophosphoethanolamines | 1.72 ± 0.76 | 7.74 ± 2.64 | 0.033 |
| PE(33:1) | 721.5 | [M+NH4]+ | PE | Diacylglycerophosphoethanolamines | 23.7 ± 3.35 | 25.2 ± 3.29 | 0.772 |
| PE(33:4) | 698.2 | [M+H]+ | PE | Diacylglycerophosphoethanolamines | 9.47 ± 2.05 | 40.7 ± 8.60 | 0.003 |
| PE(34:4) | 712.2 | [M+H]+ | PE | Diacylglycerophosphoethanolamines | 2.04 ± 0.96 | 3.84 ± 2.46 | 0.320 |
| PG(16:0) | 499.2 | [M+H]+ | PG | Diacylglycerophosphoglycerols | 15.3 ± 2.59 | 14.5 ± 2.49 | 0.842 |
| PG(28:2) | 680.4 | [M+NH4]+ | PG | Diacylglycerophosphoglycerols | 1.60 ± 0.27 | 4.02 ± 1.17 | 0.059 |
| PG(37:6) | 798.4 | [M+NH4]+ | PG | Diacylglycerophosphoglycerols | 1.31 ± 0.47 | 4.66 ± 1.72 | 0.032 |
| PS(16:0) | 529.0 | [M+NH4]+ | PS | Diacylglycerophosphoserines | 9.27 ± 1.94 | 21.5 ± 4.89 | 0.037 |
| PS(28:2) | 693.4 | [M+NH4]+ | PS | Diacylglycerophosphoserines | 9.83 ± 2.02 | 35.7 ± 8.67 | 0.011 |
| PA(O-34:2) | 659.5 | [M+H]+ | PA | 1-alkyl,2-acylglycerophosphates | 15.7 ± 3.78 | 18.2 ± 2.16 | 0.521 |
| PA(O-37:0) | 722.6 | [M+NH4]+ | PA | 1-alkyl,2-acylglycerophosphates | 42.7 ± 7.45 | 55.9 ± 7.72 | 0.209 |
| PA(O-38:1) | 734.6 | [M+NH4]+ | PA | 1-alkyl,2-acylglycerophosphates | 64.3 ± 11.3 | 141 ± 26.6 | 0.008 |
| PA(O-40:3) | 758.6 | [M+NH4]+ | PA | 1-alkyl,2-acylglycerophosphates | 53.8 ± 6.28 | 59.6 ± 6.52 | 0.510 |
| PA(O-40:3) | 758.6 | [M+NH4]+ | PA | 1-alkyl,2-acylglycerophosphates | 46.0 ± 5.19 | 52.7 ± 3.32 | 0.352 |
| PA(O-42:4) | 767.8 | [M+H]+ | PA | 1-alkyl,2-acylglycerophosphates | 3.46 ± 0.91 | 4.38 ± 0.96 | 0.229 |
| PA(P-39:1) | 746.6 | [M+NH4]+ | PA | 1-(1Z-alkenyl),2-acylglycerophosphates | 30.8 ± 2.43 | 43.4 ± 5.33 | 0.044 |
| PA(P-42:2) | 769.8 | [M+H]+ | PA | 1-(1Z-alkenyl),2-acylglycerophosphates | 39.7 ± 5.11 | 48.0 ± 2.81 | 0.159 |
| PA(P-42:4) | 782.6 | [M+NH4]+ | PA | 1-(1Z-alkenyl),2-acylglycerophosphates | 2.62 ± 0.36 | 2.83 ± 0.59 | 0.719 |
| PC(O-13:0) | 440.6 | [M+H]+ | PC | Dialkylglycerophosphocholines | 17.4 ± 1.97 | 20.7 ± 1.28 | 0.159 |
| PC(O-17:0) | 496.4 | [M+H]+ | PC | Dialkylglycerophosphocholines | 66.8 ± 11.9 | 81.5 ± 12.6 | 0.252 |
| PC(O-19:0) | 524.4 | [M+H]+ | PC | Dialkylglycerophosphocholines | 34.6 ± 3.48 | 33.6 ± 5.41 | 0.859 |
| PC(O-20:2) | 548.7 | [M+H]+ | PC | 1-alkyl,2-acylglycerophosphocholines | 20.6 ± 2.88 | 27.2 ± 3.70 | 0.181 |
| PC(O-3:0) | 317.2 | [M+H]+ | PC | Dialkylglycerophosphocholines | 23.1 ± 1.91 | 19.0 ± 1.47 | 0.114 |
| PC(O-33:2) | 747.6 | [M+NH4]+ | PC | 1-alkyl,2-acylglycerophosphocholines | 44.8 ± 6.37 | 52.3 ± 7.39 | 0.449 |
| PC(O-36:4) | 768.6 | [M+H]+ | PC | 1-alkyl,2-acylglycerophosphocholines | 78.4 ± 13.8 | 165 ± 23.1 | 0.010 |
| PC(O-37:2) | 786.8 | [M+H]+ | PC | 1-alkyl,2-acylglycerophosphocholines | 78.3 ± 12.3 | 99.7 ± 10.3 | 0.034 |
| PC(P-19:1) | 520.6 | [M+H]+ | PC | 1Z-alkenylglycerophosphocholines | 187 ± 28.8 | 189 ± 25.3 | 0.953 |
| PE(O-38:5) | 752.6 | [M+H]+ | PE | 1-alkyl,2-acylglycerophosphoethanolamines | 238 ± 32.9 | 251 ± 34.3 | 0.778 |
| PE(P-42:2) | 812.8 | [M+H]+ | PE | 1-(1Z-alkenyl),2-acylglycerophosphoethanolamines | 98.4 ± 10.1 | 96.9 ± 17.9 | 0.909 |
| PG(O-44:6) | 837.6 | [M+H]+ | PG | 1-alkyl,2-acylglycerophosphoglycerols | 2.14 ± 0.94 | 9.92 ± 4.34 | 0.092 |
| PS(O-18:0) | 683.8 | [M+NH4]+ | PS | 1-alkyl,2-acylglycerophosphoserines | 1.39 ± 0.68 | 1.68 ± 0.69 | 0.753 |
| **Lysophospholipids** |  |  |  |  |  |  |  |
| 1-linoleoyl-sn-glycero-3-phosphocholine | 520.3 | [M+H]+ | PC | Monoacylglycerophosphocholines | 20.1 ± 2.35 | 23.4 ± 3.62 | 0.383 |
| LPC(8:0) | 401.1 | [M+NH4]+ | PC | Monoacylglycerophosphocholines | 6.24 ± 1.26 | 15.4 ± 4.32 | 0.055 |
| LPC(10:0) | 429.4 | [M+NH4]+ | PC | Monoacylglycerophosphocholines | 17.9 ± 2.68 | 23.4 ± 4.34 | 0.274 |
| LPC(12:0) | 440.4 | [M+H]+ | PC | Monoacylglycerophosphocholines | 21.0 ± 1.46 | 22.9 ± 4.07 | 0.564 |
| LPC(14:1) | 466.4 | [M+H]+ | PC | Monoacylglycerophosphocholines | 16.3 ± 1.48 | 18.7 ± 2.84 | 0.529 |
| LPC(15:0) | 499.4 | [M+NH4]+ | PC | Monoacylglycerophosphocholines | 15.1 ± 3.06 | 16.6 ± 2.61 | 0.738 |
| LPC(16:1) | 494.4 | [M+H]+ | PC | Monoacylglycerophosphocholines | 16.7 ± 2.22 | 15.2 ± 2.50 | 0.710 |
| LPC(18:0) | 541.8 | [M+NH4]+ | PC | Monoacylglycerophosphocholines | 7.72 ± 0.94 | 15.2 ± 4.14 | 0.130 |
| LPC(18:1) | 539.3 | [M+NH4]+ | PC | Monoacylglycerophosphocholines | 23.9 ± 2.29 | 26.4 ± 4.51 | 0.565 |
| LPC(18:1) | 539.6 | [M+NH4]+ | PC | Monoacylglycerophosphocholines | 11.3 ± 0.98 | 11.7 ± 2.30 | 0.837 |
| LPC(19:0) | 555.4 | [M+NH4]+ | PC | Monoacylglycerophosphocholines | 2.33 ± 0.78 | 1.86 ± 0.91 | 0.547 |
| LPC(24:0) | 625.6 | [M+NH4]+ | PC | Monoacylglycerophosphocholines | 10.8 ± 2.43 | 27.0 ± 6.86 | 0.023 |
| LPC(O-10:1) | 413.2 | [M+H]+ | PC | Monoalkylglycerophosphocholines | 17.9 ± 1.49 | 16.7 ± 1.81 | 0.621 |
| LPC(O-16:1) | 497.4 | [M+NH4]+ | PC | Monoalkylglycerophosphocholines | 84.9 ± 10.1 | 87.6 ± 12.6 | 0.790 |
| LPE(15:1) | 438.2 | [M+H]+ | PE | Monoacylglycerophosphoethanolamines | 6.51 ± 1.51 | 16.0 ± 4.83 | 0.078 |
| LPE(16:1) | 469.7 | [M+NH4]+ | PE | Monoacylglycerophosphoethanolamines | 5.66 ± 1.21 | 19.0 ± 4.69 | 0.013 |
| LPE(18:2) | 495.4 | [M+NH4]+ | PE | Monoacylglycerophosphoethanolamines | 23.3 ± 3.82 | 35.8 ± 4.55 | 0.036 |
| LPE(20:3) | 504.1 | [M+H]+ | PE | Monoacylglycerophosphoethanolamines | 18.2 ± 1.93 | 20.7 ± 4.05 | 0.514 |
| LPE(20:4) | 519.4 | [M+NH4]+ | PE | Monoacylglycerophosphoethanolamines | 1.66 ± 0.65 | 1.61 ± 0.50 | 0.902 |
| LPG(16:1) | 500.4 | [M+NH4]+ | PG | Monoacylglycerophosphoglycerols | 1.78 ± 0.16 | 1.74 ± 0.27 | 0.883 |
| LPG(20:1) | 556.4 | [M+NH4]+ | PG | Monoacylglycerophosphoglycerols | 32.0 ± 5.27 | 36.8 ± 6.10 | 0.348 |
| LPG(20:4) | 533.1 | [M+H]+ | PG | Monoacylglycerophosphoglycerols | 9.24 ± 1.95 | 22.2 ± 5.04 | 0.031 |
| LPG(21:0) | 572.5 | [M+NH4]+ | PG | Monoacylglycerophosphoglycerols | 6.52 ± 1.18 | 18.1 ± 5.11 | 0.045 |
| LPG(22:1) | 584.6 | [M+NH4]+ | PG | Monoacylglycerophosphoglycerols | 41.5 ± 5.13 | 46.6 ± 8.88 | 0.596 |
| LPI(12:0) | 533.9 | [M+NH4]+ | PI | Monoacylglycerophosphoinositols | 20.1 ± 2.74 | 21.3 ± 2.29 | 0.759 |
| LPS(13:0) | 473.4 | [M+NH4]+ | PS | Monoacylglycerophosphoserines | 22.4 ± 3.35 | 27.0 ± 4.17 | 0.252 |
| LPS(14:0) | 487.2 | [M+NH4]+ | PS | Monoacylglycerophosphoserines | 24.9 ± 2.28 | 21.7 ± 3.25 | 0.395 |
| LPS(18:0) | 526.5 | [M+H]+ | PS | Monoacylglycerophosphoserines | 22.9 ± 2.10 | 26.3 ± 5.11 | 0.460 |
| LPS(22:4) | 591.5 | [M+NH4]+ | PS | Monoacylglycerophosphoserines | 21.3 ± 3.06 | 30.1 ± 5.10 | 0.102 |
| LPS(O-20:0) | 540.6 | [M+H]+ | PS | Monoalkylglycerophosphoserines | 40.3 ± 12.8 | 120 ± 31.0 | 0.037 |
| **Sphingolipids** |  |  |  |  |  |  |  |
| Cer(30:2) | 480.4 | [M+H]+ | Ceramides | N-acylsphingosines (ceramides) | 17.4 ± 2.22 | 17.5 ± 1.74 | 0.983 |
| Cer(32:3) | 506.6 | [M+H]+ | Ceramides | N-acylsphingosines (ceramides) | 22.5 ± 3.18 | 22.9 ± 3.52 | 0.940 |
| Cer(33:2) | 522.8 | [M+H]+ | Ceramides | N-acylsphingosines (ceramides) | 14.2 ± 1.73 | 14.6 ± 1.45 | 0.827 |
| Cer(36:1) | 583.6 | [M+NH4]+ | Ceramides | N-acylsphingosines (ceramides) | 26.2 ± 2.69 | 33.8 ± 4.20 | 0.105 |
| Cer(36:3) | 579.6 | [M+NH4]+ | Ceramides | N-acylsphingosines (ceramides) | 15.4 ± 1.94 | 19.8 ± 2.01 | 0.155 |
| Cer(37:2) | 578.8 | [M+H]+ | Ceramides | N-acylsphingosines (ceramides) | 18.7 ± 0.81 | 28.7 ± 3.92 | 0.043 |
| Cer(38:1) | 627.6 | [M+NH4]+ | Ceramides | N-acylsphingosines (ceramides) | 23.9 ± 1.97 | 37.5 ± 4.21 | 0.042 |
| Cer(38:1) | 611.6 | [M+NH4]+ | Ceramides | N-acylsphingosines (ceramides) | 26.3 ± 3.46 | 33.4 ± 5.00 | 0.287 |
| Cer(38:3) | 607.6 | [M+NH4]+ | Ceramides | N-acylsphingosines (ceramides) | 18.9 ± 2.06 | 23.2 ± 3.54 | 0.341 |
| Cer(40:1) | 638.6 | [M+H]+ | Ceramides | N-acylsphingosines (ceramides) | 17.5 ± 2.92 | 22.3 ± 2.27 | 0.219 |
| Cer(42:1) | 650.7 | [M+H]+ | Ceramides | N-acylsphingosines (ceramides) | 32.2 ± 4.85 | 31.9 ± 5.41 | 0.965 |
| Cer(43:1) | 664.7 | [M+H]+ | Ceramides | N-acylsphingosines (ceramides) | 61.8 ± 6.27 | 63.9 ± 9.18 | 0.841 |
| Cer(34:0) | 556.6 | [M+H]+ | Ceramides | N-acyl-4-hydroxysphinganines | 18.8 ± 1.59 | 21.4 ± 2.25 | 0.335 |
| Cer(38:0) | 596.6 | [M+H]+ | Ceramides | N-acyl-4-hydroxysphinganines | 25.1 ± 2.63 | 24.5 ± 3.19 | 0.905 |
| Cer(34:0) | 540.6 | [M+H]+ | Ceramides | N-acylsphinganines (dihydroceramides) | 20.7 ± 3.39 | 22.4 ± 3.74 | 0.728 |
| Cer(34:0) | 589.6 | [M+NH4]+ | Ceramides | N-acylsphinganines (dihydroceramides) | 88.3 ± 20.8 | 128 ± 14.3 | 0.041 |
| Cer(36:0) | 568.6 | [M+H]+ | Ceramides | N-acylsphinganines (dihydroceramides) | 1.05 ± 0.15 | 1.09 ± 0.22 | 0.822 |
| Cer(36:0) | 601.6 | [M+NH4]+ | Ceramides | N-acylsphinganines (dihydroceramides) | 21.1 ± 2.53 | 20.7 ± 3.11 | 0.860 |
| Cer(38:0) | 645.6 | [M+NH4]+ | Ceramides | N-acylsphinganines (dihydroceramides) | 20.9 ± 2.76 | 19.8 ± 1.90 | 0.784 |
| Cer(40:0) | 641.6 | [M+NH4]+ | Ceramides | N-acylsphinganines (dihydroceramides) | 38.9 ± 7.17 | 46.9 ± 5.23 | 0.322 |
| Cer(40:0) | 624.8 | [M+H]+ | Ceramides | N-acylsphinganines (dihydroceramides) | 26.2 ± 3.35 | 38.6 ± 5.27 | 0.030 |
| Cer(46:0) | 725.8 | [M+NH4]+ | Ceramides | N-acylsphinganines (dihydroceramides) | 44.4 ± 6.47 | 51.7 ± 5.72 | 0.319 |
| PE-Cer(38:2) | 748.6 | [M+NH4]+ | Phosphosphingolipids | Ceramide phosphoethanolamines | 3.05 ± 0.74 | 3.54 ± 1.29 | 0.736 |
| PE-Cer(40:2) | 760.8 | [M+NH4]+ | Phosphosphingolipids | Ceramide phosphoethanolamines | 279 ± 69.8 | 402 ± 75.3 | 0.079 |
| SM(34:2) | 718.6 | [M+NH4]+ | Phosphosphingolipids | Ceramide phosphocholines (sphingomyelins) | 71.8 ± 14.3 | 97.4 ± 8.37 | 0.047 |
| SM(36:3) | 744.6 | [M+NH4]+ | Phosphosphingolipids | Ceramide phosphocholines (sphingomyelins) | 110 ± 19.4 | 147 ± 21.4 | 0.208 |
| SM(38:2) | 774.8 | [M+NH4]+ | Phosphosphingolipids | Ceramide phosphocholines (sphingomyelins) | 40.3 ± 5.58 | 44.1 ± 6.68 | 0.613 |
| SM(39:2) | 788.8 | [M+NH4]+ | Phosphosphingolipids | Ceramide phosphocholines (sphingomyelins) | 146 ± 22.8 | 211 ± 10.0 | 0.041 |
| SM(42:2) | 813.8 | [M+H]+ | Phosphosphingolipids | Ceramide phosphocholines (sphingomyelins) | 43.7 ± 5.64 | 50.1 ± 6.76 | 0.420 |
| Sphingosine(14:2) | 242.2 | [M+H]+ | Sphingoid bases | Sphingoid base analogs | 6.85 ± 0.74 | 9.67 ± 1.13 | 0.033 |
| Sphingosine(18:3) | 296.4 | [M+H]+ | Sphingoid bases | Sphingoid base analogs | 131 ± 34.6 | 245 ± 30.3 | 0.011 |
| C16 Sphingosine | 289.2 | [M+NH4]+ | Sphingoid bases | Sphingoid base homologs and variants | 12.3 ± 2.35 | 15.8 ± 2.28 | 0.093 |
| C17 Sphinganine | 305.4 | [M+NH4]+ | Sphingoid bases | Sphingoid base homologs and variants | 5.65 ± 0.48 | 4.84 ± 0.90 | 0.420 |
| Sphinganine-1-phosphocholine | 484.4 | [M+NH4]+ | Sphingoid bases | Lysosphingomyelins and lysoglycosphingolipids | 64.6 ± 15.0 | 42.2 ± 6.73 | 0.242 |
| **Sterols** |  |  |  |  |  |  |  |
| 15:0 Cholesteryl ester | 628.8 | [M+NH4]+ | Sterols | Steryl esters | 22.5 ± 1.22 | 33.0 ± 5.93 | 0.128 |
| 16:0 Cholesteryl ester | 625.6 | [M+H]+ | Sterols | Steryl esters | 19.8 ± 1.00 | 21.0 ± 3.05 | 0.714 |
| 16:2 Cholesteryl ester | 638.8 | [M+NH4]+ | Sterols | Steryl esters | 63.0 ± 7.02 | 58.8 ± 13.7 | 0.817 |
| 16:3 Cholesteryl ester | 636.6 | [M+NH4]+ | Sterols | Steryl esters | 34.0 ± 5.93 | 26.3 ± 4.59 | 0.255 |
| 18:2 Cholesteryl ester | 666.8 | [M+NH4]+ | Sterols | Steryl esters | 102 ± 14.6 | 124 ± 20.1 | 0.363 |
| 19:0 Cholesteryl ester | 684.8 | [M+NH4]+ | Sterols | Steryl esters | 43.0 ± 9.46 | 56.3 ± 8.06 | 0.308 |
| 22:3 Cholesteryl ester | 720.8 | [M+NH4]+ | Sterols | Steryl esters | 21.0 ± 3.33 | 35.9 ± 2.03 | 0.001 |
| 22:4 Cholesteryl ester | 718.8 | [M+NH4]+ | Sterols | Steryl esters | 18.2 ± 1.88 | 20.0 ± 2.53 | 0.591 |
| 24,25-dihydrolanosterol | 429.4 | [M+H]+ | Sterols | Cholesterol and derivatives | 15.9 ± 1.91 | 13.1 ± 2.59 | 0.393 |
| 24,25-epoxy-cholesterol | 418.4 | [M+NH4]+ | Sterols | Cholesterol and derivatives | 19.4 ± 1.32 | 12.2 ± 1.47 | 0.001 |
| 24-hydroxy-cholesterol | 420.4 | [M+NH4]+ | Sterols | Cholesterol and derivatives | 17.0 ± 0.86 | 13.3 ± 1.88 | 0.076 |
| 3,5-cholestadien-7-one | 400.4 | [M+NH4]+ | Sterols | Cholesterol and derivatives | 18.3 ± 0.62 | 24.3 ± 2.20 | 0.038 |
| 4,4-dimethylcholesta-8,11,24-trienol | 428.6 | [M+NH4]+ | Sterols | Cholesterol and derivatives | 24.4 ± 1.36 | 24.3 ± 3.66 | 0.989 |
| 4β-(hydroxymethyl)-4α-methyl-5α-cholest-7-en-3β-ol | 431.4 | [M+H]+ | Sterols | Cholesterol and derivatives | 23.7 ± 2.43 | 27.3 ± 3.59 | 0.506 |
| 6α-hydroxycholestanol | 422.6 | [M+NH4]+ | Sterols | Cholesterol and derivatives | 18.5 ± 1.57 | 12.3 ± 2.75 | 0.083 |

Values are mean signal intensity (million units) ± SEM. P-value from paired, two-tailed t-Student’s test.
